# Influence of Modic changes on short-term clinical and patient-reported outcomes after anterior cervical discectomy and fusion: a retrospective cohort study

**DOI:** 10.3389/fneur.2026.1926807

**Published:** 2026-09-11

**Authors:** Mustafa Emrah Kaya, Ali Maksut Aykut, İbrahim Barışcan Soydan, Mahmut Cingöz, Mustafa Aras, Yurdal Serarslan, Şükrü Oral

**Affiliations:** 1 Department of Neurosurgery, Faculty of Medicine, Hatay Mustafa Kemal University, Antakya, Hatay, Türkiye; 2 Department of Radiology, Faculty of Medicine, Hatay Mustafa Kemal University, Antakya, Hatay, Türkiye; 3 Department of Neurosurgery, Faculty of Medicine, Ondokuz Mayıs University, Samsun, Türkiye; 4 Department of Neurosurgery, Faculty of Medicine, Ordu University, Ordu, Türkiye; 5 Department of Neurosurgery, Kayseri City Hospital, Kocasinan, Kayseri, Türkiye

**Keywords:** anterior discectomy and fusion, cervical spine, intervertebral disc degeneration, Modic changes, Neck Disability Index, patient-reported outcomes, spinal surgery

## Abstract

**Background:**

Modic changes are MR signal changes in the vertebral endplate and adjacent subchondral bone marrow that reflect degenerative and inflammatory processes. Their prognostic significance after anterior discectomy and fusion (ACDF) remains incompletely defined.

**Objective:**

To determine whether Modic changes are associated with baseline disease severity and short-term clinical and patient-reported outcomes after ACDF.

**Methods:**

We retrospectively analyzed 130 patients with classifiable Modic status who underwent ACDF and completed 6-month follow-up. The primary outcome measure was the 6-month change in the Neck Disability Index (NDI). Secondary outcomes included neck and arm Visual Analog Scale (VAS) scores, motor grade, and clinically important responder rates. Comparisons were made using appropriate parametric or non-parametric tests. Multivariable, propensity-score-adjusted, and inverse-probability-of-treatment-weighted analysis were used to evaluate the independent association between Modic status and NDI improvement.

**Results:**

Thirty-six patients were Modic-positive, and 94 were Modic-negative. Modic-positive patients were older (52.3 vs. 45.9 years; *p* = 0.011). Additionally, these patients exhibited a higher surgical level Pfirrmann grade (4.39 vs. 4.02; *p* < 0.001), greater spinal cord compression (*p* = 0.001), and a higher prevalence of myelomalacia (44.4% vs. 21.3%; *p* = 0.015). Significant improvements in neck VAS, arm VAS, NDI, and motor grade scores were observed in both groups at the 6-month mark (all within-group *p* < 0.001). Although post-procedure residual neck VAS and NDI scores were higher in Modic-positive patients, the change scores did not differ significantly between the groups. Modic status was not found to be independently associated with NDI improvement in the expanded multivariable model (*β* = 0.90; 95% CI, −0.44 to 2.24; *p* = 0.185), the propensity-score-adjusted model (*β* = 0.98; 95% CI, −2.15 to 4.11; *p* = 0.537), or the weighted analysis (*β* = 0.25; 95% CI, −2.65 to 3.15; *p* = 0.865). NDI responder rates were 91.7 and 83.0%, respectively (*p* = 0.328).

**Conclusion:**

Cervical Modic changes were associated with more advanced structural degeneration, spinal cord compression, and myelomalacia, but they did not show an independent association with the level of short-term improvement following ACDF. Modic status alone should not be considered an adverse short-term prognostic factor in appropriately selected patients.

## 1 Introduction

Modic changes (MCs) are signal intensity changes visible on magnetic resonance imaging (MRI) in the vertebral body end plate and adjacent subchondral bone marrow. Initially, they were classified into three types. Type 1 is edematous or inflammatory; it appears hypointense on T1-weighted images and hyperintense on T2-weighted images. Type 2 reflects fatty bone marrow replacement, appearing hyperintense in T1 and iso-hyperintense in T2. Type 3 is sclerotic and appears hypointense in both sequences (1). MCs are now widely accepted as imaging correlates of a degenerative and low-grade inflammatory cascade involving the disc and end plate complex (2, 3). Their pathobiology is characterized by end plate damage, persistent inflammation, high bone turnover, and fibrosis, and the three types are thought to represent interdependent stages of a single underlying process (4, 5).

Lumbar spine MCs have been associated with discogenic pain and, in some series, with less favorable conservative and surgical outcomes. The cervical spine has received far less attention. A recent systematic review and meta-analysis of over 12,000 participants confirmed that cervical MCs are common, with reported prevalence varying widely across populations, and concluded that their prognostic significance for surgery is not adequately defined (6). Pooled analyses also suggest that cervical MCs are associated with disc degeneration and longer-term pain rather than symptom severity itself (7) and reflect a degenerative process in the endplate (8). Contemporary surgical series add nuance to this picture. After anterior cervical discectomy and fusion (ACDF), patients with MCs report higher preoperative neck pain and disability and show a higher rate of cage subsidence and impaired fusion, but their functional recovery is generally comparable to that of patients without MCs (9). Similar concerns have been raised regarding a more complex radiological course, including heterotopic ossification, following cervical disc arthroplasty in the presence of MCs (10). In combined cervical and lumbar surgery cohorts, patients with MCs nevertheless show a similar degree of significant improvement after surgery as patients without MCs (11).

Whether cervical MCs simply reflect further degeneration or independently alter the clinical course after surgical decompression remains unclear and clinically significant. The answer is directly related to patient counseling and surgical decision-making. We conducted a retrospective cohort study on patients undergoing cervical disc surgery with two aims. The first was to characterize how Modic-positive and Modic-negative patients differed in terms of baseline clinical and radiological severity. The second was to determine whether the presence of MCs was associated with the magnitude of clinical and patient-reported improvement at 6 months before and after correction for relevant covariates.

## 2 Materials and methods

### 2.1 Study design and population

**Figure 1:**
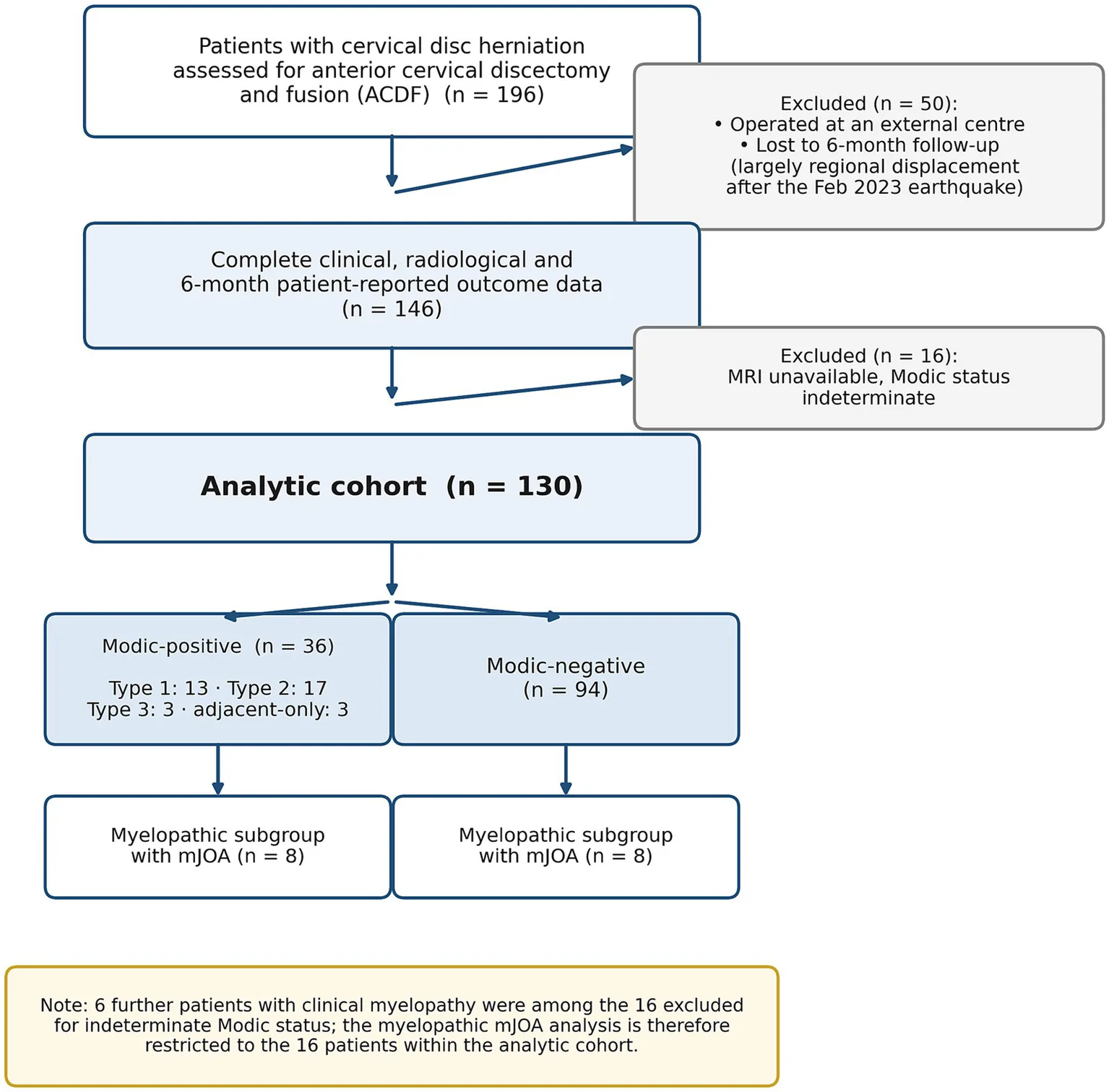
STROBE flow diagram of patient selection. Consecutive patients undergoing anterior cervical discectomy and fusion (ACDF) were screened according to the study eligibility criteria. The final analytical group consisted of 130 patients with a classifiable Modic status and complete clinical, radiological, and 6-month follow-up data. ACDF, anterior cervical discectomy and fusion; mJOA, modified Japanese Orthopaedic Association; MRI, magnetic resonance imaging.

This single-center retrospective cohort study included consecutive patients who underwent ACDF for symptomatic cervical degenerative disc disease presenting with radiculopathy, myelopathy, or both. During the study period, 196 patients were evaluated for eligibility. Of these, 50 patients were excluded because they had undergone surgery at another institution or did not complete the scheduled 6-month follow-up, primarily owing to regional displacements following the February 2023 earthquake. The remaining 146 patients had completed clinical, radiological, and follow-up data. A further 16 patients were excluded because preoperative magnetic resonance imaging (MRI) was unavailable, preventing classification of Modic changes. Consequently, the final analysis cohort consisted of 130 patients (Figure 1).

The study was conducted in accordance with the Declaration of Helsinki and approved by the Hatay Mustafa Kemal University Clinical Research Ethics Committee (Decision No. 67, dated 06/05/2026). Due to the retrospective design, the requirement for written informed consent was waived.

### 2.2 Surgical treatment

All patients underwent a standard anterior cervical discectomy and fusion (ACDF) procedure performed by experienced neurosurgeons under general anesthesia. Microsurgical discectomy was carried out via a standard right-sided anterior cervical approach following fluoroscopic confirmation of the target level. The cartilaginous endplates were meticulously prepared while preserving the bony endplates whenever possible. Following decompression of the spinal cord and nerve roots (including opening of the posterior longitudinal ligament when necessary), an appropriately sized bladed PEEK interbody cage with integrated ceramic titanium-alloy blades, packed with synthetic bone graft putty, was inserted under fluoroscopic guidance. All procedures were performed by one of four experienced neurosurgeons who were trained at the same institution and used a standard surgical technique. Wound closure was performed in anatomical layers. The surgical level(s) were determined based on the correlation between neurological findings, clinical symptoms, and MRI findings. The number of treated levels for each patient was recorded and included in the statistical analyses.

### 2.3 Variables and outcome measures

Primary variables included age, sex, symptom duration, number of operated levels, and comorbidity. Exposure of interest was the presence of one or more Modic changes (Modic positive and Modic negative). When graded, the Modic type at the surgical level (type 1, 2, or 3) was also recorded, with a value of 0 indicating no change at that level. Radiological severity was determined by the Pfirrmann disc degeneration grade (out of 5) recorded at the surgical level and as a global cervical mean (12), graded cord compression ratio, and presence of myelomalacia.

Clinical outcomes were assessed preoperatively and 6 months later. The primary outcome was the change in Neck Disability Index (NDI; range 0–50) from baseline to 6 months (13). Secondary outcomes included changes in neck and arm pain measured using the Visual Analog Scale (VAS; range, 0–10), motor grade, and, in patients with clinical myelopathy, the modified Japanese Orthopaedic Association (mJOA; range, 018) (14). Change scores were calculated by subtracting the preoperative value from the 6-month value. Therefore, negative change scores for the VAS and NDI indicated clinical improvement.

### 2.4 Imaging acquisition and grading

**Figure 2:**
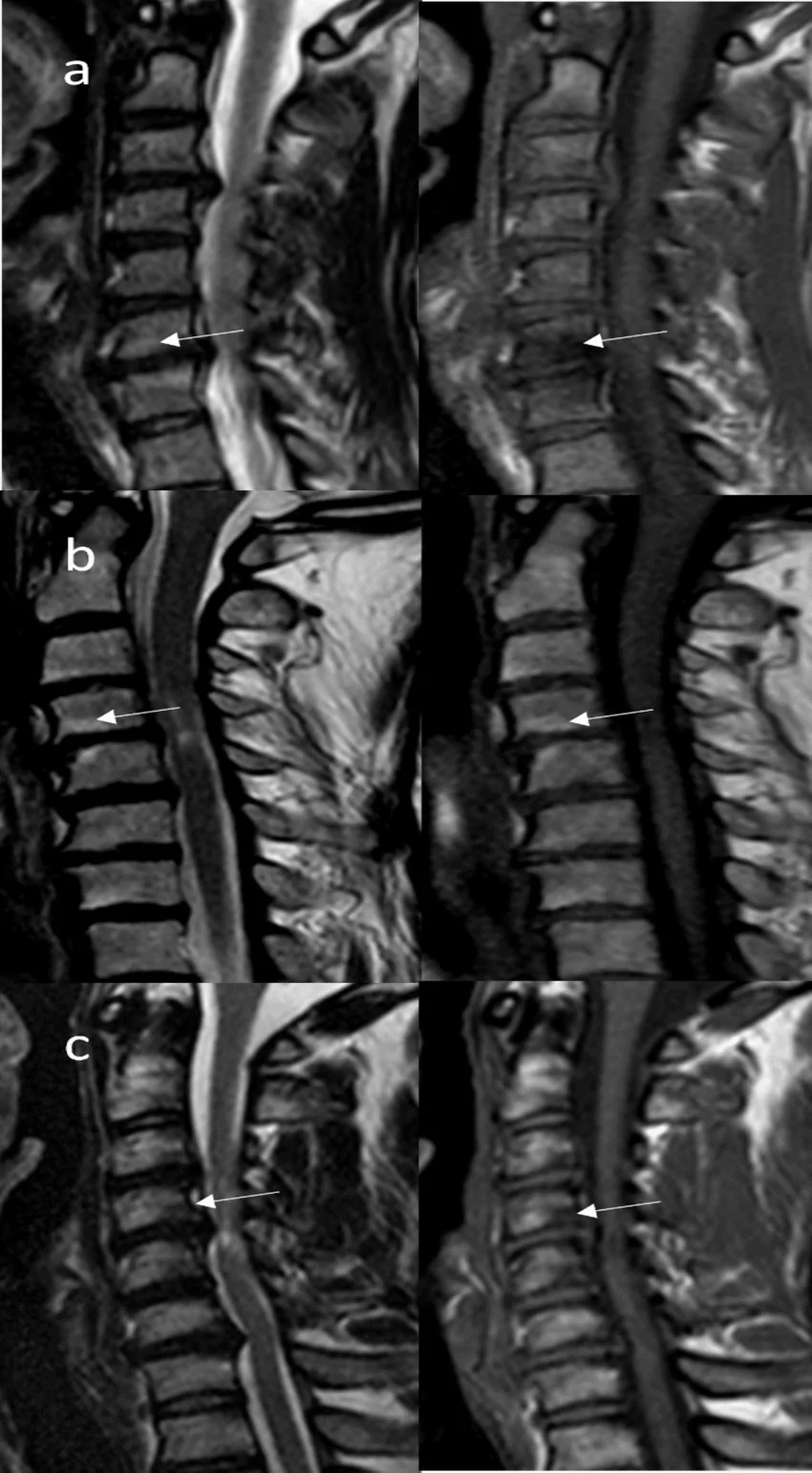
Representative cervical Modic changes. Mid-sagittal T2-weighted images (left column) and corresponding T1-weighted images (right column) showing **(a)** Modic type 1, **(b)** Modic type 2, and **(c)** Modic type 3 changes (arrows).

**Figure 3:**
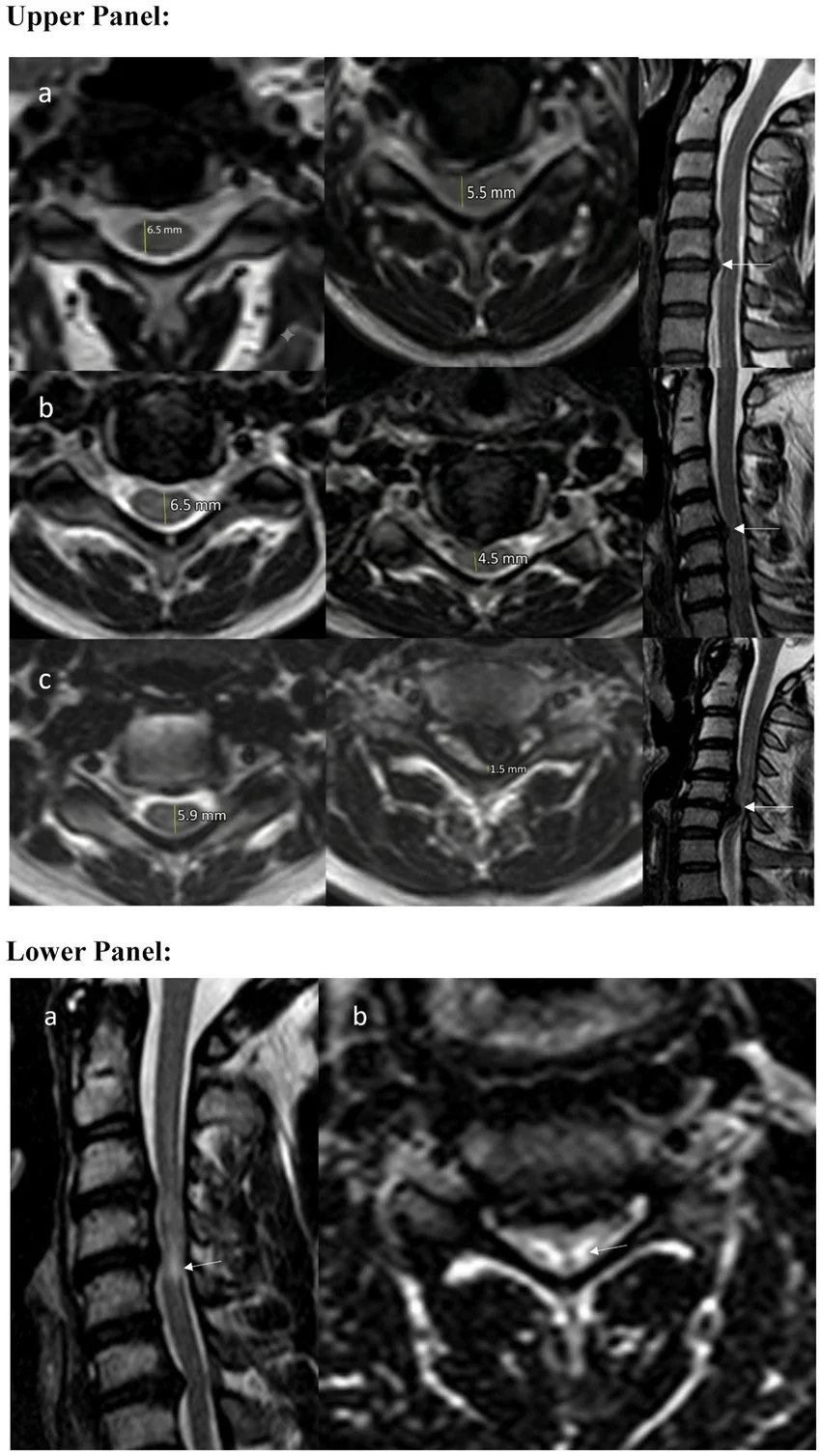
Measurement of spinal cord compression and representative myelomalacia. *Upper panel*: Representative axial and corresponding sagittal T2-weighted MR images from three patients **(a–c)** illustrating assessment of spinal cord compression. For each patient, the anteroposterior diameter of the spinal cord was measured at the level of maximal compression and compared with that at a normal, uncompressed level to calculate the spinal cord compression ratio. Compression severity was subsequently classified as mild or absent (0.8–1.0), moderate (0.5–0.8), or severe (<0.5). *Lower panel*: Representative sagittal and axial T2-weighted MR images (arrows) showing intramedullary T2 hyperintensity consistent with cervical myelomalacia.

**Figure 4:**
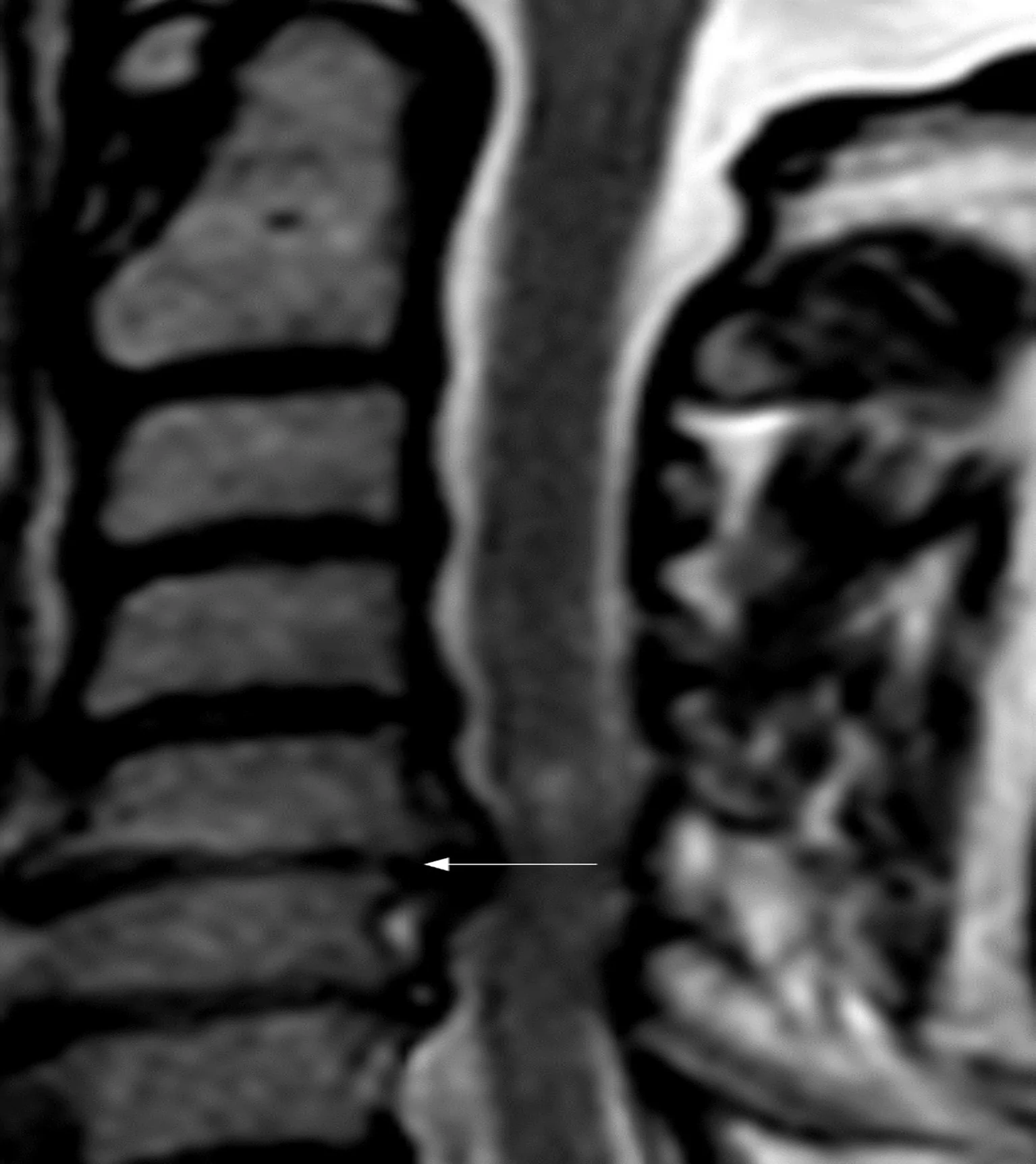
Representative Pfirrmann grade 5 cervical disc degeneration. Mid-sagittal T2-weighted MRI showing severe degeneration at the C5–6 level with associated Modic changes. The operated level exhibits a higher Pfirrmann grade compared with the adjacent discs.

**Figure 5:**
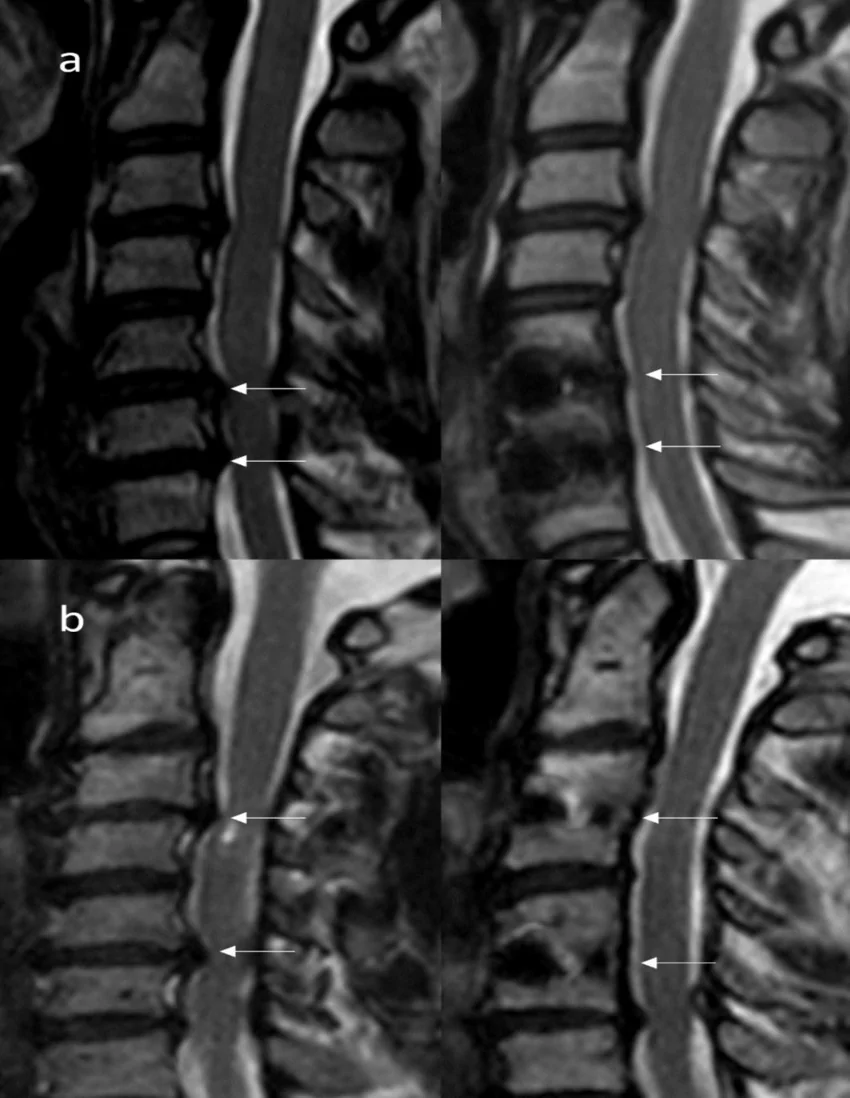
Representative preoperative and postoperative MRI findings. Sagittal T2-weighted MR images obtained before (left) and after (right) surgery in representative patients with **(a)** Modic changes and **(b)** without Modic changes. Arrows indicate the surgical level, demonstrating satisfactory decompression of the spinal cord following ACDF.

All patients underwent cervical spine MRI using the institution’s standard protocol. Modic changes were defined as endplate and subchondral bone marrow signal changes and classified as type 1 (T1 hypointense, T2 hyperintense), type 2 (T1 hyperintense, T2 iso- to hyperintense), or type 3 (T1 and T2 hypointense) following the original definition (1). Disc degeneration was graded at the surgical level using the Pfirrmann classification (out of 5), with the arithmetic mean taken among the operated discs in multi-level cases and determined as the global cervical mean at all levels (12). Spinal cord compression ratio was measured as the ratio of the anteroposterior spinal cord diameter at the compressed level (A) to the diameter at the normal, uncompressed level (B) in the same patient, on sagittal or axial T2-weighted images at the maximal compression level. The A/B ratio was classified as mild or absent (0.8–1.0), moderate (0.5–0.8), or severe (below 0.5) and recorded as grades 1, 2, and 3, respectively; in multi-level cases, the average of the level-specific ratios was used. Myelomalacia was recorded as the presence of intramedullary T2 hyperintensity. Figures 2, 3, 4, 5 illustrate representative examples of Modic changes, spinal cord compression measurement, cervical myelomalacia, severe disc degeneration (Pfirrmann grade 5), and postoperative spinal cord decompression. All images were graded by two radiologists co-authors, who read the studies independently and blinded to clinical outcomes. Their ratings were concordant, and any discrepancies were resolved by consensus.

### 2.5 Statistical analysis

Continuous variables were summarized as mean (standard deviation) and median. The primary outcome was the change in NDI score from baseline to the 6-month follow-up. Normality of distribution was assessed using the Shapiro–Wilk test. Intergroup comparisons were made using the Welch *t*-test for approximately normal variables, otherwise the Mann–Whitney *U* test was used. Categorical variables were compared using the chi-square or Fisher exact test, as appropriate. Within-group changes from baseline to 6 months were tested using the Wilcoxon signed-rank test. In the myelopathic subgroup, the Hirabayashi recovery rate was calculated by dividing postoperative minus preoperative mJOA by 18 minus preoperative mJOA, expressed as a percentage, and classified as excellent (above 75%), good (50–74%), moderate (25–49%), or poor (below 25%) (15). Each continuous comparison was accompanied by a standardized effect size (Cohen *d*). To evaluate whether Modic status independently predicted improvement, each score of change was modeled with Modic status using a multivariate linear regression model, considering age, gender, symptom duration, and operated levels. To further evaluate the robustness of the findings, additional sensitivity analyses were conducted using propensity-score-adjusted regression and stabilized inverse probability of treatment weighting (IPTW). A two-sided *p*-value below 0.05 was considered statistically significant. Analyses were performed in Python (pandas, SciPy, and statsmodels). As this was a retrospective study, no *a priori* sample size calculation was performed. A *post hoc* analysis indicated that the available samples (36 vs. 94) provided 80% power at an *α* = 0.05 (two-sided) to detect a between-group difference of 3.8 NDI points. This difference was below the accepted minimal clinically important difference of approximately 7.5 points.

## 3 Results

### 3.1 Cohort characteristics

Of 130 patients, 36 (27.7%) were Modic positive and 94 (72.3%) were Modic negative. The mean age of the overall cohort was 47.7 years (range 22–78), 53.8% were female, and they were mostly treated at one (65.4%) or two (31.5%) levels. Patients with Modic changes were significantly older than those without Modic changes (52.3 vs. 45.9 years, *p* = 0.011), whereas gender, symptom duration, number of operated levels, comorbidity, and complication rates were comparable between the groups. Detailed baseline demographic, clinical, and radiological characteristics are summarized in Table 1. Disc herniation was classified as protrusion in 122 patients, bulging in 6, and extrusion in 2 patients.

**Table 1:** Baseline characteristics according to Modic change status.

Characteristic	Modic + (*n* = 36)	Modic − (*n* = 94)	*p* value
Age, mean (SD), years	52.3 (13.1)	45.9 (9.8)	0.011
Female gender, *n* (%)	16 (44.4)	54 (57.4)	0.257
Symptom duration, mean (SD), months	18.3 (14.9)	21.8 (20.2)	0.688
Number of operated level, mean (SD)	1.42 (0.55)	1.36 (0.55)	0.556
Any comorbidity, *n* (%)	9 (25.0)	19 (20.2)	0.722
Clinical myelopathy at presentation, *n* (%)	8 (22.2)	8 (8.5)	0.069
Complications, *n* (%)	0 (0.0)	5 (5.3)	0.322

SD, standard deviation. *p* values from Welch *t*-test (continuous) or chi-square/Fisher exact test (categorical) results. Modic-positive indicates the presence of one or more Modic changes at the operated or adjacent cervical level, whereas Modic-negative indicates no detectable Modic changes.

### 3.2 Radiological severity

**Figure 6:**
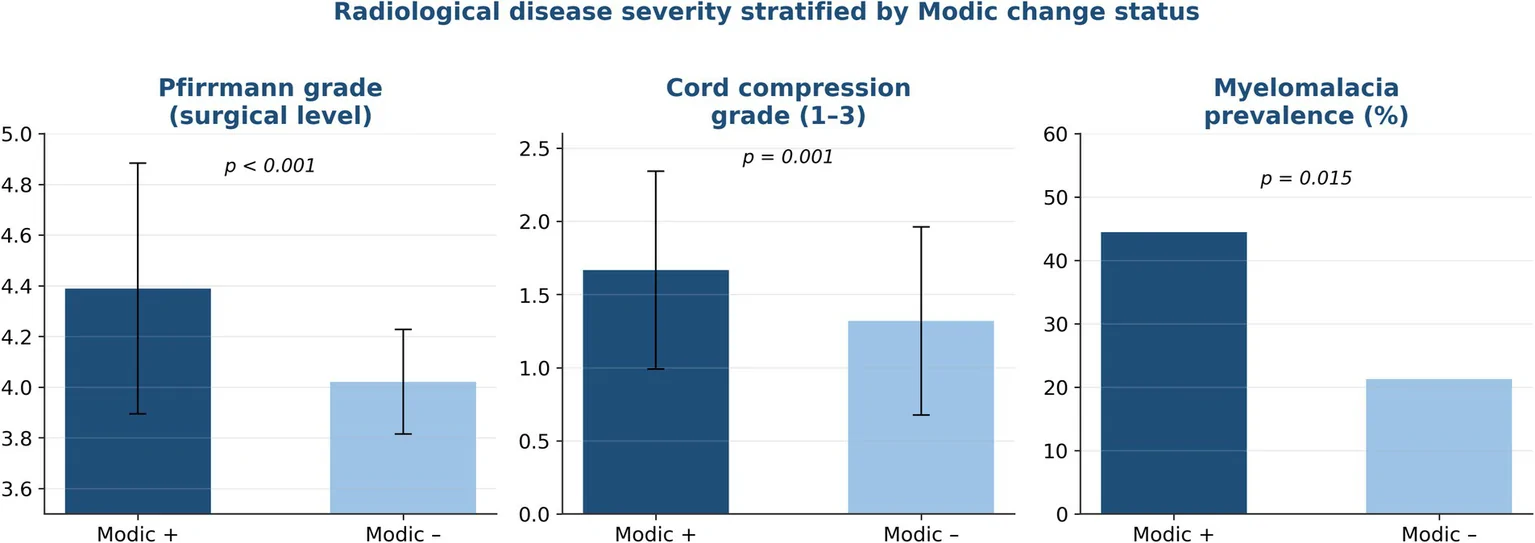
Radiological disease severity according to Modic status. In Modic-positive patients, a higher Pfirrmann grade at the surgical level, a higher rate of spinal cord compression, and a higher prevalence of myelomalacia were observed.

Patients with Modic changes demonstrated more advanced radiological degeneration than those without Modic changes. The Pfirrmann grade at the surgical level was significantly higher (4.39 vs. 4.02, *p* < 0.001), with a large standardized effect size (Cohen *d* = 1.18). The spinal cord compression grade was higher (1.67 vs. 1.32, *p* = 0.001) and myelomalacia was approximately twice as common (44.4% vs. 21.3%, *p* = 0.015). The overall Pfirrmann mean showed only a non-significant difference between groups (*p* = 0.069; Table 2, Figure 6). Of the 36 patients with Modic changes, 17 (47.2%) had type 2 changes at the surgical level, 13 (36.1%) had type 1 and 3 (8.3%) had type 3, whereas in 3 patients (8.3%) the Modic changes were confined to an adjacent level.

**Table 2:** Radiological severity according to Modic status.

Radiological variable	Modic +	Modic −	*p* value
Pfirrmann grade, surgical level, mean (SD)	4.39 (0.49)	4.02 (0.21)	<0.001
Pfirrmann grade, global, mean (SD)	4.03 (0.17)	3.96 (0.20)	0.069
Spinal cord compression grade (1–3), mean (SD)	1.67 (0.68)	1.32 (0.64)	0.001
Myelomalacia, *n* (%)	16 (44.4)	20 (21.3)	0.015

*p*-values from Mann–Whitney *U* test (continuous) or Chi-square or Fisher’s exact test, as appropriate.

### 3.3 Clinical and patient-reported outcomes

**Figure 7:**
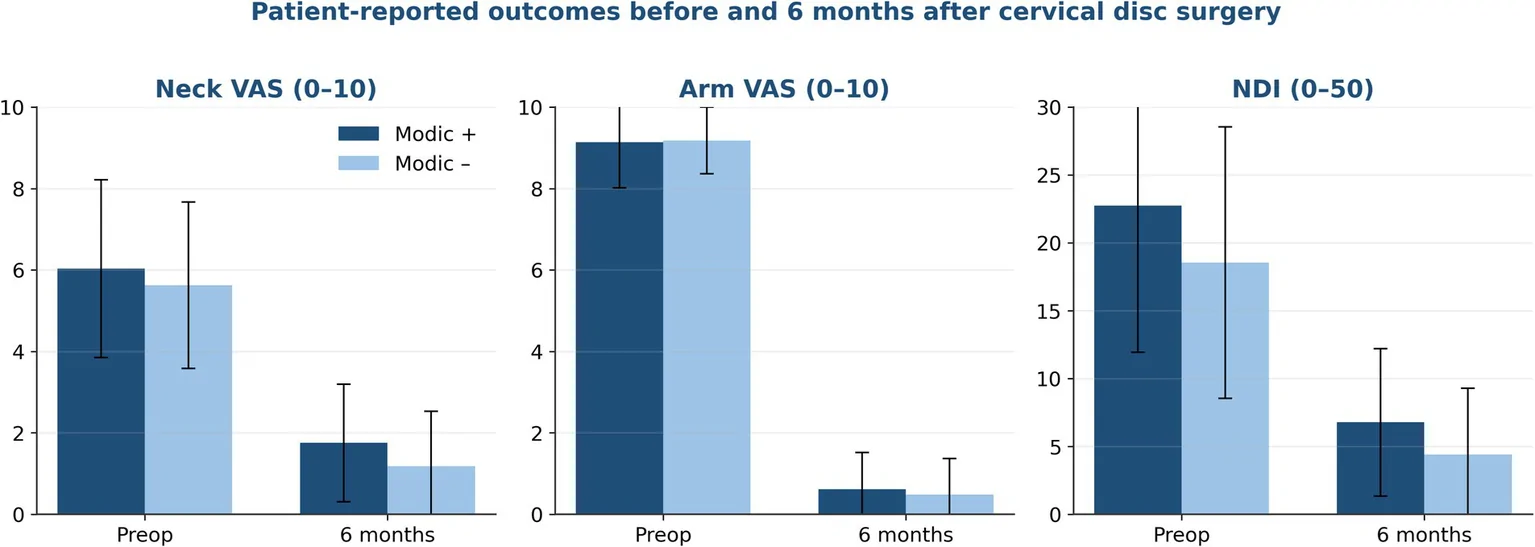
Patient-reported outcomes before and 6 months after cervical disc surgery, stratified according to Modic status. Bars show mean and standard deviation. Both groups showed highly significant improvement across Neck VAS, Arm VAS, and NDI (all within-group *p* < 0.001).

Both groups showed significant improvement at the 6-month follow-up. In within-group analyses, neck VAS, arm VAS, NDI, and motor grade improved significantly from baseline in both Modic-positive and Modic-negative groups (all *p* < 0.001, Figure 7). Overall, at 6 months, 95.4% of patients had reached a level of no disability or mild disability (NDI ≤ 14), while 49.2% had reached a level of no disability (NDI ≤ 4).

**Figure 8:**
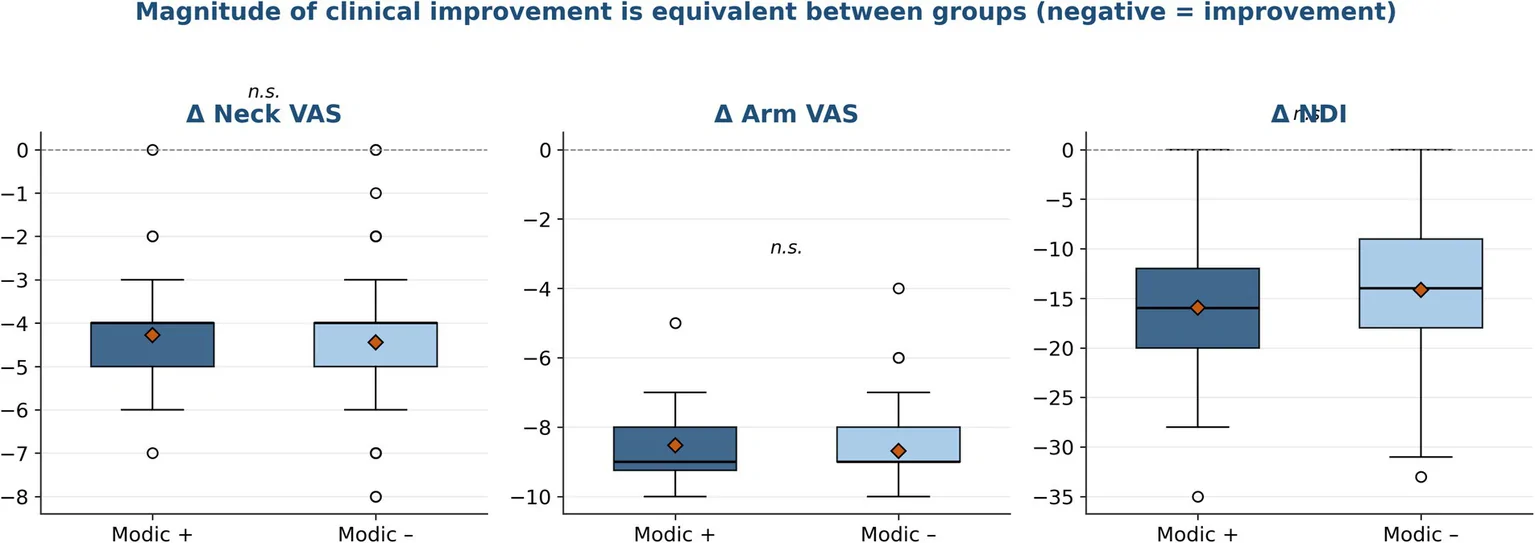
Distribution of change scores according to Modic status. Orange diamonds indicate the group mean, and negative values represent improvement. The magnitude of improvement did not differ significantly between groups. n.s., not significant.

Patients with Modic changes presented with a greater baseline symptom burden, reflected by a higher preoperative NDI score than patients without Modic changes (22.7 vs. 18.5, *p* = 0.048). At 6 months, residual neck VAS (1.75 vs. 1.18, *p* = 0.025) and NDI (6.78 vs. 4.40, *p* = 0.019) remained slightly higher in the Modic-positive group. However, the magnitude of improvement (change scores) did not differ significantly between groups for neck VAS (*p* = 0.643), arm VAS (*p* = 0.469), or NDI (*p* = 0.178), and the corresponding effect sizes were small (Table 3, Figure 8).

**Table 3:** Preoperative, 6-month, and change scores according to Modic status.

Outcome measure	Modic + mean (SD)	Modic − mean (SD)	Effect size (Cohen *d*)	*p* value
Preop neck VAS	6.03 (2.18)	5.63 (2.04)	0.19	0.321
6-month neck VAS	1.75 (1.44)	1.18 (1.34)	0.42	0.025
Change in neck VAS	−4.28 (1.28)	−4.45 (1.53)	0.12	0.643
Preop arm VAS	9.14 (1.13)	9.18 (0.82)	−0.05	0.617
6-month arm VAS	0.61 (0.90)	0.49 (0.88)	0.14	0.490
Change in arm VAS	−8.53 (1.21)	−8.69 (1.03)	0.15	0.469
Preop NDI	22.7 (10.8)	18.5 (10.0)	0.41	0.048
6-month NDI	6.78 (5.44)	4.40 (4.87)	0.47	0.019
Change in NDI	−15.9 (6.6)	−14.1 (7.1)	−0.26	0.178
Preop motor grade	3.94 (1.07)	4.23 (1.04)	−0.28	0.114
6-month motor grade	4.72 (0.45)	4.83 (0.38)	−0.27	0.173

VAS, Visual Analog Scale (0–10). NDI, Neck Disability Index (0–50). Negative change scores indicate greater clinical improvement. *p* values from Mann–Whitney *U* or Welch *t*-test.

Responder analyses likewise showed no significant difference between the groups. An improvement in the NDI meeting or exceeding the minimal clinically important difference (MCID) was achieved in 91.7% of Modic-positive patients and 83.0% of Modic-negative patients (*p* = 0.328). Similarly, responder rates based on MCID thresholds for neck and arm VAS were high in both groups, and no significant difference was found between the groups.

### 3.4 Myelopathic subgroup

The final analysis cohort included 16 patients (8 Modic-positive and 8 Modic-negative) who had clinical myelopathy with available mJOA. Six additional patients with clinical myelopathy were excluded from this subgroup analysis because preoperative MRI was unavailable, preventing classification of Modic status (Figure 1).

Substantial neurological recovery was observed postoperatively in both groups. The mean mJOA score improved from 9.1 to 15.9 in the Modic-positive group and from 9.4 to 15.2 in the Modic-negative group. These findings correspond to Hirabayashi recovery rates of 76.6 and 67.6%, respectively. The difference between the groups was not statistically significant (*p* = 0.366). Overall, 15 of the 16 patients (93.8%) achieved a good or excellent Hirabayashi recovery. Given the small sample size of the subgroups, these findings should be considered exploratory and interpreted with caution.

### 3.5 Adjusted analysis

To determine whether Modic status independently influenced postoperative recovery, multivariate linear regression analyses were performed using change scores for NDI, neck VAS, and arm VAS as dependent variables, after adjusting for age, sex, duration of symptoms, and the number of operated levels. Modic status did not show an independent association with improvement in NDI (adjusted *β* = −1.60; 95% CI −3.97 to 0.78; *p* = 0.186), neck VAS (adjusted *β* = −0.05; 95% CI −0.62 to 0.53; *p* = 0.876), or arm VAS (adjusted *β* = −0.02; 95% CI −0.45 to 0.42; *p* = 0.942) values (Table 4).

**Table 4:** Multivariable linear regression analysis of the association between Modic status and postoperative improvement.

Outcome (change score)	Adjusted *β* coefficient	95% CI	*p*-value
Changes in NDI	−1.60	−3.97 to 0.78	0.186
Changes in Neck VAS	−0.05	−0.62 to 0.53	0.876
Changes in Arm VAS	−0.02	−0.45 to 0.42	0.942

Models were adjusted for age, sex, symptom duration, and number of operated levels. *β* represents the adjusted coefficient for Modic-positive versus Modic-negative status.

Additional sensitivity analyses using an expanded multivariable model, propensity score adjustment, and stabilized inverse probability of treatment weighting (IPTW) yielded consistent findings, with no independent association between Modic status and postoperative NDI improvement across any model (Supplementary Table S1). No statistically significant association was found between Modic status and postoperative recovery in any of the models. This supports the robustness of the primary analyses.

### 3.6 Outcomes by Modic type

Among patients with Modic changes, exploratory analyses were conducted to compare clinical outcomes between Modic type 1 and type 2 lesions at the surgical level. The type 3 subgroup (3 patients) was too small for a meaningful comparison and was not tested. No statistically significant difference was observed between type 1 and type 2 in either baseline symptom severity or postoperative improvement (Table 5). Patients with Modic type 1 and type 2 lesions demonstrated similar clinical improvement across all evaluated outcome measures. This suggests that the Modic subtype does not significantly influence short-term surgical outcomes in this patient group.

**Table 5:** Comparison of outcomes between Modic type 1 and Modic type 2 lesions at the surgical level.

Outcome measure	Type 1 (*n* = 13), mean (SD)	Type 2 (*n* = 17), mean (SD)	Cohen *d*	*p* value
Preop NDI	24.6 (9.2)	22.4 (10.4)	0.23	0.516
Change in NDI	−16.5 (5.2)	−15.4 (6.0)	−0.20	0.502
Preop neck VAS	6.62 (1.89)	5.82 (2.01)	0.40	0.318
Change in neck VAS	−4.46 (1.13)	−4.06 (0.83)	−0.42	0.212
Preop arm VAS	9.54 (0.97)	9.00 (1.17)	0.49	0.135
Change in arm VAS	−8.62 (1.19)	−8.53 (1.01)	−0.08	0.829

Negative change values indicate improvement. *p*-values from the Mann–Whitney *U* test. Type 3 subgroup (*n* = 3) was excluded from comparative analysis because of insufficient sample size.

## 4 Discussion

In this retrospective cohort of 130 patients undergoing anterior cervical discectomy and fusion (ACDF), cervical Modic changes were associated with a more advanced degenerative phenotype rather than diminished short-term surgical benefit. Modic-positive patients were older and had more advanced segmental degeneration with higher Pfirrmann grades, more spinal cord compression, and more frequent myelomalacia; preoperative and postoperative symptom burden was also slightly higher. Nevertheless, the principal finding is reassuring: The magnitude of clinical improvement did not differ significantly between groups, and Modic status did not independently predict postoperative improvement after adjustment for age, sex, symptom duration, and number of operated levels. The prevalence of Modic changes in our study group (27.7%) is consistent with current findings. Nezameslami et al. reported considerable variability among published cervical patient groups; however, they found that Modic changes were seen in approximately one quarter of surgically treated patients, which places our findings within the expected range (6). Similarly, Gao et al. also reported a comparable prevalence in an observational group of cervical patients (16).

This pattern reconciles two observations that might otherwise seem contradictory. Modic-positive patients finished the follow-up period with slightly worse absolute neck VAS and NDI scores. At first glance, this may suggest a worse prognosis. However, because they started from a worse baseline, their net gain from surgery matched that of Modic-negative patients. Moreover, despite slightly higher residual symptom scores in the Modic-positive group, the overall clinical outcome was favorable in both groups. At 6 months, 95.4% of the cohort reached the no-disability or mild-disability range (NDI ≤ 14), while 49.2% reached the no-disability range (NDI ≤ 4), according to the original NDI categories (13). This finding further supports the effectiveness of cervical decompression regardless of Modic status. The clinically relevant message is that Modic changes identify a more advanced degenerative phenotype but do not diminish the clinical benefit achieved with surgical decompression.

A particularly striking finding is the strong association between Modic changes and myelomalacia. Myelomalacia was approximately twice as common in Modic-positive segments (44.4% vs. 21.3%, *p* = 0.015) and was accompanied by a higher degree of spinal cord compression. These findings suggest that Modic changes may serve not only as indicators of vertebral endplate degeneration but also as imaging markers of a spinal cord environment associated with a higher risk of chronic neurological compromise. Whereas previous studies on cervical Modic changes have primarily focused on outcomes related to pain, disability, and fusion, there has been limited attention to the relationship between Modic changes and spinal cord signaling abnormalities, making this finding particularly noteworthy (6). This finding is consistent with evidence indicating that Modic changes are markers of advanced cervical degeneration and endplate failure (17), as well as with the proposed pathobiology of these changes, which involves endplate damage, inflammation, and increased bone turnover (4). The coexistence of marrow-signal and intramedullary cord signal change may mark segments where the degenerative cascade has reached the cord itself. Importantly, this condition did not result in a poorer recovery process; indeed, in a study of 518 patients undergoing ACDF, Oris et al. reported that preoperative myelomalacia did not lead to worse clinical outcomes, noting that baseline scores and demographic factors, rather than the myelomalacia itself, were the primary determinants of success (18). Thus, while the sustained improvement observed in our patients with Modic changes and myelomalacia is reassuring, it also suggests that this phenotype may warrant closer and long-term monitoring.

These results are broadly consistent with current cervical literature. Our findings are align Deng and colleagues who showed that patients with cervical Modic changes undergoing ACDF had more advanced degeneration and less favorable radiographic outcomes, including greater subsidence, but their postoperative functional recovery remained generally comparable (9). The predominance of Modic type 2 in our cohort is also consistent with previous cervical studies and systematic reviews, in which fatty endplate degeneration represents the most frequently encountered Modic subtype in patients undergoing surgery (6). This likely reflects the chronic nature of cervical degenerative disc disease in surgical populations. Similarly, despite the increased risk of heterotopic ossification, satisfactory clinical outcomes have been reported after cervical disc arthroplasty in patients with Modic changes. This strengthens the role of Modic changes in patient selection rather than being a contraindication (10). Nezameslami et al.’s systematic review reported a strong association between cervical Modic changes and disc degeneration, with an inconsistent and generally small independent effect on surgical outcome (6), and pooled surgery cohorts also show a similar improvement to patients without Modic changes (11). Broader meta-analytic data suggest that cervical Modic changes are associated with pain duration rather than symptom severity; this is consistent with the modest residual symptom difference we observed (7, 8). In patients with predominantly neck pain, both fusion and arthroplasty provide significant disability improvement, highlighting that residual scores are driven by the baseline symptom profile and not Modic changes status alone (19). The concentration of the degenerative burden at the surgical level in our data, namely a significant surgical-level Pfirrmann difference but only a global trend, supports a localized disc and endplate process rather than a widespread cervical phenotype.

Taken together, these findings suggest that Modic changes should be interpreted primarily as markers of structural disease severity rather than as predictors of diminished surgical benefit. The lack of a significant difference between groups reflects a lack of detectable effect rather than proven equivalence. However, the primary comparison was adequately powered to detected an NDI difference of 3.8 points (below the MCID of roughly 7.5 points), which strengthens this conclusion.

**Table:** 

*Clinical takeaway*. The presence of Modic changes alone should not deter ACDF. Modic-positive patients can be counseled that they are likely to achieve short-term recovery comparable to Modic-negative patients, while being advised that more advanced baseline degeneration may leave marginally higher residual symptom scores.

## 5 Limitations

The study has several limitations that should be considered. First, although the selection process is documented in the STROBE flow diagram (Figure 1), the retrospective, single-center design entails inherent risks of selection and information bias. Second, the follow-up period was limited to 6 months. Effects associated with Modic changes on fusion, subsidence, adjacent segment degeneration, and the durability of symptom relief may only become apparent over a period of 1–5 years (9, 20, 21), and a longer-term analysis of this cohort is planned. Third, radiographic assessment was restricted to fusion and Modic status, as pre-specified. Consequently, subsidence, alignment, cervical lordosis, adjacent segment degeneration, and reoperation status were not systematically recorded. Fourth, since only consensus-based imaging assessments were utilized, a numerical inter-observer agreement coefficient (kappa or intraclass correlation) could not be calculated. Nevertheless, two radiologists performed independent, blinded ratings (without knowledge of clinical data), yielding consistent results. Fifth, variables such as smoking status, diabetes, and body mass index were not recorded and thus could not be included in the analysis. Sixth, Modic changes were primarily analyzed based on their presence or absence. While Type 2 changes were predominant, the Type 3 subgroup was small (3 patients), precluding a statistically powered comparison between types. Similarly, the myelopathic subgroup with available mJOA data (16 patients) was also limited in size. Finally, no sample size calculation was performed prior to the study. A *post hoc* analysis demonstrated 80% statistical power to detect an NDI difference of 3.8 points, which was below the minimum clinically important difference, Therefore, while the primary comparison was adequately powered, the subgroup analyses regarding Modic type and myelopathy remained exploratory in nature.

## 6 Conclusion

Cervical Modic changes are associated with more advanced degenerative disease characterized by greater segmental degeneration, more severe spinal cord compression, and a higher prevalence of myelomalacia. Despite these differences, Modic status was not independently associated with the magnitude of short-term clinical improvement after anterior cervical discectomy and fusion. Patients with Modic changes experienced improvements in pain, disability, neurological function, and patient-reported outcomes comparable to those observed in patients without Modic changes. Therefore, in appropriately selected patients, Modic changes alone should not be considered an adverse prognostic factor or a contraindication to surgical decompression. Larger prospective studies with longer follow-up are warranted to clarify their influence on long-term radiological and clinical outcomes.

## Data Availability

The datasets presented in this study can be found in online repositories. The names of the repository/repositories and accession number(s) can be found in the article/Supplementary material.
