# Supplementary material for: Influence of Modic changes on short-term clinical and patient-reported outcomes after anterior cervical discectomy and fusion: a retrospective cohort study
Source: Front Neurol. 2026 Sep 11;17:1926807. doi: 10.3389/fneur.2026.1926807 (PMC13612358; doi:10.3389/fneur.2026.1926807)
Supplement: Supplementary file 1 [file Table_1.docx]

**Supplementary Table S1**. Effect of Modic status on the 6-month change in the Neck Disability Index (primary outcome) across four adjustment strategies.

| **Adjustment model** | **β** | **95% CI** | **p** |
| --- | --- | --- | --- |
| Base multivariable | −1.60 | −3.97 to 0.78 | 0.186 |
| Expanded multivariable | 0.90 | −0.44 to 2.24 | 0.185 |
| Propensity score-adjusted | 0.98 | −2.15 to 4.11 | 0.537 |
| Stabilized IPTW | 0.25 | −2.65 to 3.15 | 0.865 |

Across four adjustment strategies, the 95% confidence intervals included zero, indicating no consistent independent association between Modic status and the magnitude of NDI improvement.
